# Validation of selective use of intraoperative PTH monitoring in parathyroidectomy

**DOI:** 10.1186/s40463-017-0188-0

**Published:** 2017-02-06

**Authors:** Alexandra Thielmann, Paul Kerr

**Affiliations:** Department of Otolaryngology-Head and Neck Surgery, University of Manitoba, Winnipeg, Manitoba Canada

**Keywords:** Primary hyperparathyroidism, Parathyroidectomy, Parathyroid hormone, Technetium Tc 99 m sestamibi

## Abstract

**Background:**

The objective of this study was to validate our approach of treating primary hyperparathyroidism using sestamibi scan directed parathyroidectomy, without routine use of intraoperative parathyroid hormone measurements (ioPTH).

**Methods:**

We prospectively established a protocol limiting the use of ioPTH to patients with negative or equivocal sestamibi scans, and those who had risk factors for multi-gland disease. We then performed a retrospective review to determine our disease control rate.

**Results:**

128 patients underwent sestamibi-guided parathyroidectomy without (111/128 = 87%) or with (17/128 = 13%) ioPTH. The overall disease control (eucalcemia) rate was 95%. 3/111 (3%) of patients who had surgery without ioPTH measurements required re-exploration.

**Conclusions:**

Selective use of ioPTH is an effective strategy. ioPTH is best reserved for patients who have non-localizing preoperative imaging, are at risk for multi-gland disease, or require revision surgery.

## Background

Surgical treatment of primary hyperparathyroidism (PHPT) has greatly evolved over the past couple of decades. What was once a tedious four-gland exploration has become a relatively short procedure directed at the solitary abnormal gland in most instances. This paradigm shift has been facilitated by improved preoperative localization, usually with a Tc 99 m sestamibi scan with or without ultrasound, and the advent of intraoperative testing with intraoperative parathyroid hormone levels or a gamma probe.

Intraoperative PTH monitoring (ioPTH) is by far the most common adjunct used during surgery. Proponents of routine ioPTH utilization site the advantage of immediate confirmation of successful treatment at the time of surgery, and the ability to detect multi- gland disease. Multi-gland disease is purported to occur in as many as 15% of cases, which would seem to support the use of routine testing.

The potential disadvantages of routine ioPTH utilization are cost, added operating time, and the possibility of false results that prompt further, unnecessary exploration. Therefore, one can make the argument for selective utilization. The rate of multi-gland disease is very small in properly selected patients who have definitive localizing scans [1–3].

We prospectively established a protocol of directed, uni-glandular surgery based on preoperative localization, limiting the use of ioPTH to patients with negative or equivocal sestamibi scans, and those who had risk factors for multi-gland disease. The objective of this study is to review our rates of ioPTH utilization and disease control using this protocol.

## Methods

The Department of Otolaryngology-Head and Neck Surgery, University of Manitoba, established a protocol in 2009 for selective use of ioPTH in the surgical management of PHTP. All patients would undergo preoperative localization using Tc 99 m sestamibi scanning with a view to attempting directed, uni-glandular surgery when possible. Ultrasound was used to strengthen preoperative localization in selected cases. Use of ioPTH was reserved for patients with negative or equivocal preoperative localization, and/or risk factors for multi-glandular disease such as known multiple endocrine neoplasia or a positive family history.

This protocol ultimately created two groups of patients:

- Group 1: Abnormal gland localized; surgeon anticipates probable single adenoma and plans directed, uni-glandular surgery without ioPTH
- Group 2: Abnormal gland not localized; surgeon anticipates increased risk for multi-gland disease or smaller adenoma, plans for high likelihood of 4 gland exploration, and arranges ioPTH

Frozen section pathology was used to confirm the nature of the tissue removed intraoperatively in all patients. Three fellowship trained head and neck surgeons performed all the operations, with the level of experience ranging from less than 5 years in practice to greater than 15 years in practice. All procedures were performed in tertiary care institutions with a high level of radiology and pathology support.

We performed a retrospective chart review of patients treated with this protocol between 2009 and 2014. The Bannatyne Campus Research Ethics Board approved this study. We initially included all patients undergoing surgery for primary hyperparathyroidism, with or without ioPTH monitoring. Data collected included patient demographics, preoperative workup, operative findings, and postsurgical outcomes.

In reviewing preoperative workup, we collected data on preoperative calcium and parathyroid hormone (PTH) levels, and imaging performed (sestamibi and ultrasound). The imaging outcomes were categorized as either positive, or negative (including weak, discordant, or ambiguous results). Operative findings mainly focused on the use of ioPTH, however we included documenting bilateral and unilateral number and intraoperative pathology of glands removed.

Postoperatively we collected pathology reports, operative reports, and calcium and PTH levels. Blood results were available in the provincial electronic laboratory reporting system, enabling biochemical follow-up often long after the completion of surgical follow-up. A successful operation was defined as median post operation calcium levels below the high limit of normal. Failure was stringently defined as ANY calcium levels above normal following surgery associated with an inappropriately elevated PTH level.

In the case of those requiring re-operation, we further investigated these charts for qualitative information pertaining to the surgical procedure and circumstances surrounding the failure.

## Results

140 consecutive patients underwent parathyroidectomy over the course of this study. Twelve patients were excluded due to a variety of issues: lack of follow-up data; confounding conditions such as systemic malignancy; patient did not complete sestamibi scan or scan results were not available.

This left 128 patients in the analysis, 90 females and 38 males. The mean age was 60, with a range of 20–89 years. Median follow-up was 16 months (range 1–67 months). The mean preoperative corrected calcium level was 2.9 +/− 0.3 mmol/l. The mean preoperative PTH level was 183 +/− 232 ng/l.

**Fig. 1:**
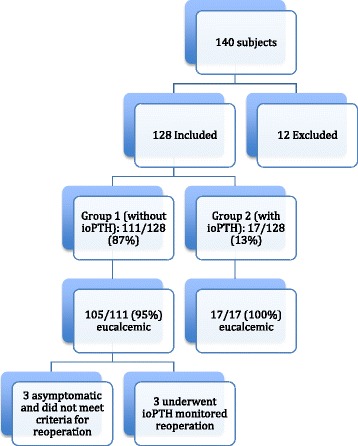
Patient Selection and Outcomes

111/128 (87%) underwent radiologically guided parathyroidectomy without ioPTH (Group 1), and 17/128 (13%) with ioPTH (Group 2) (Fig. 1). All patients underwent sestamibi scanning preoperatively. 32/128 (25%) had both sestabmibi and ultrasound. 106/111 (95%) Group 1 patients were managed with unilateral exploration. The majority (11/17 (65%)) of Group 2 patients required bilateral exploration, as anticipated.

The overall failure rate, by very strict criteria (median elevated calcium levels with inappropriately PTH, documented at any time postoperatively) was 6/128 (5%). Only 3/128 required re-exploration (2%). These 3 patients were subsequently rendered eucalcemic. The other 3 cases that were classified as failures were in fact significantly improved but had very mild, asymptomatic residual hypercalcemia that did not meet NIH criteria for further surgery.

The failures were within Group 1 (no ioPTH) . Thus the failure rate for sestamibi-guided surgery without ioPTH was 6/111 (5%), and the re-exploration rate was 3/111 (3%).

## Discussion

There is a good body of literature supporting the use of ioPTH monitoring. The addition of ioPTH has facilitated a transition to radiologically-guided, minimally invasive parathyroid surgery. This has improved surgical outcomes relative to traditional four-gland exploration both by decreasing operative times, hospital costs, and avoiding unnecessary risk to the patient [4–7]. Despite this, routine use of ioPTH for primary hyperparathyroidism surgery may be unnecessary in appropriately selected patients.

Proponents of routine use of ioPTH quote multi-gland disease figures upwards of 15% [8–11]. However, the rate of multi-gland disease in patients that have no risk factors, and a positive preoperative localization is likely far less than that. The low rate of failure after uni-glandular surgery in our study and others [1, 3, 12] would support the notion that the rate of multi-gland disease in properly selected patients is 5% or less.

The authors acknowledge the importance of analyzing cost-effectiveness in any debate regarding one treatment vs another; in this case routine use vs selective use of ioPTH in parathyroidectomy for primary hyperparathyroidism. However, the retrospective nature of this study precludes any sophisticated analysis of cost differential and is beyond the design of the project. Nevertheless, we feel that a basic comment on a clinical impression that is apparent in many centers, and that provides the impetus for a movement away from routine use of ioPTH is warranted: Waiting 10–20 min to draw blood and the subsequent wait for a ioPTH assay adds clinically significant amounts of time to surgery when compared to sestimibi directed surgery with frozen section confirmation that an enlarged parathyroid has been removed.

The first 10–20 min after adenoma excision in either case represents wound closure and simultaneous waiting for frozen section (non-ioPTH case) or time to pass before drawing the ioPTH bloodwork (ioPTH protocol). The subsequent wait for an ioPTH assay often adds over 15–30 min to the case in our institution. Therefore, one can roughly calculate that 25–50 h of operating time would be consumed for every 100 cases, awaiting ioPTH results. Considering this would have been for theoretically reducing our reoperation rate from 3 patients per hundred down to 0–2 per hundred, this hardly seems worthwhile.

Even if prospectively collected randomized cost-effectiveness data pertaining to the extended time and cost for one protocol vs another is available, one must consider many other issues that are much more difficult to factor into the analysis: What are the effects of freeing up 25–50 h of operating on patients OUTSIDE the study? What is the cost of unnecessarily extended operations based on misleading results in the ioPTH group? What is the cost of reoperation of patients who have persistent disease in both groups? What is the QALY impact of complications from over-exploration in the ioPTH group? What is the QALY impact of uncontrolled disease in both groups?

We, like many centers, rely on sestamibi with or without ultrasound for accurate localization. Some studies have shown up to 97–100% sensitivity and 100% specificity of sestamibi scans [13–15]. Factors shown to decrease sensitivity or result in negative scans are multi-gland disease, small hypersecreting glands, lower levels of serum calcium, and body mass index and gland size [16–19]. These factors can easily be determined clinically, allowing confident selection of patients for ioPTH.

Our results are consistent with similar published studies investigating image guided parathyroidectomy without ioPTH, ranging from success rates of 92–100% [20–22]. Comparatively, our local failure rate was 5% with a reoperation rate of 3%. Jacobson et al. [1] published a similar retrospective analysis in 2002, reporting eucalcemia in 97% with persistent hypercalcemia in 3 patients (3%), one of which required reoperation. They note similar inclusion criteria as in our study. In another more recent study in 2015, Mownah et al. cite a 97% cure rate without ioPTH in patients with concordant preoperative sestamibi and ultrasound [2]. In this instance on-table ultrasound was used. In contrast, a review published in 2015, stresses the need for ioPTH monitoring to avoid surgical failure [23], however, their success rates were 94.9% in patients without ioPTH and 100% with ioPTH. Failure of imaging to localize the adenoma or discordance between sestamibi and ultrasound imaging was used to determine use of ioPTH, again similar to our protocol. While the success rate in this study is an impressive 100%, success rates in the non-ioPTH group still fall within expected ranges cited above.

Proper patient selection for ioPTH does not guarantee a successful outcome, as even minimally invasive surgery with ioPTH monitoring can lead to failures. One in fifteen patients in our study remained persistently hypercalcemic with a negative follow-up sestamibi. Ultimately, the gold standard has been bilateral neck exploration by an experienced head and neck surgeon. In 2004 Siperstein et al. found additional pathology in the contralateral neck in 15% of patients with ioPTH monitoring and concordant sestamibi and ultrasound imaging [24]. Surgeon experience led to bilateral exploration in patients with two abnormal appearing glands. Later, a review of the usefulness of ioPTH in 2011 concluded that the test may only be as good as the surgeon, requiring proper interpretation considering possible multi-gland disease and using appropriate ioPTH criteria [25].

With such conflicting opinions, it is interesting that Sitger-Serra et al. note that using technical advances, such as ioPTH may be more appealing to less experienced surgeons, as it may serve as a fail-safe or compensate for anatomical intricacies of parathyroid surgery [26]. It is obviously important for centers to consider local expertise and the availability and cost of timely ioPTH testing when developing local strategies for minimizing the cost and time of parathyroidectomy, and the need for re-exploration.

One might be tempted to conclude that our zero-failure rate in the non-localizing group, in which ioPTH was used, contradicts our support for exploration without ioPTH. However, it is important to note that this is a small group of 17 patients. Obviously, with an adequate sample size the failure rate will exceed zero. As well, these 17 patients were managed not only with the addition of ioPTH, but also frequent bilateral exploration, additional imaging, and additional OR time. Therefore, one cannot conclude that it is the addition of ioPTH that is the sole factor that may have improved outcome in this group. To use all of these additional measures in every sestimibi localized case would go entirely against the recognized success and savings of directed exploration, and potentially add risk in terms of unnecessarily extended exploration.

Philosophically, one must accept the fact that there will be a very small percent of cases that may have to return to the operating room for further exploration no matter how much preoperative testing, intraoperative testing, and exploration is performed. If one really wants to reduce the risk of needing re-exploration as much as possible, one would have to add ioPTH and other forms of imaging to routine four-gland exploration which will result in an unreasonable increase in cost, OR time and risk.

## Conclusions

Selective use of ioPTH is an effective strategy. Intraoperative PTH monitoring is best reserved for patients who have non-localizing preoperative imaging, are at risk for multi-gland disease, or require revision surgery.

## Abbreviations

ioPTH: Intraoperative parathyroid hormone
PHTP: Primary hyperparathyroidism
PTH: Parathyroid hormone.
